# Microdevice Platform for In Vitro Nervous System and Its Disease Model

**DOI:** 10.3390/bioengineering4030077

**Published:** 2017-09-13

**Authors:** Jin-Ha Choi, Hyeon-Yeol Cho, Jeong-Woo Choi

**Affiliations:** 1 Department of Chemical & Biomolecular Engineering, Sogang University, 35 Baekbeom-ro, Mapo-Gu, Seoul 04107, Korea; jinhachoi@sogang.ac.kr (J.-H.C.); yeol@sogang.ac.kr (H.-Y.C.); 2 Department of Chemistry and Chemical Biology, Rutgers, The State University of New Jersey, 610 Taylor Road, Piscataway, NJ 08854, USA

**Keywords:** organ-on-a-chip, nervous system-on-a-chip, neuronal interaction, neurodegenerative disease, stem cell

## Abstract

The development of precise microdevices can be applied to the reconstruction of in vitro human microenvironmental systems with biomimetic physiological conditions that have highly tunable spatial and temporal features. Organ-on-a-chip can emulate human physiological functions, particularly at the organ level, as well as its specific roles in the body. Due to the complexity of the structure of the central nervous system and its intercellular interaction, there remains an urgent need for the development of human brain or nervous system models. Thus, various microdevice models have been proposed to mimic actual human brain physiology, which can be categorized as nervous system-on-a-chip. Nervous system-on-a-chip platforms can prove to be promising technologies, through the application of their biomimetic features to the etiology of neurodegenerative diseases. This article reviews the microdevices for nervous system-on-a-chip platform incorporated with neurobiology and microtechnology, including microfluidic designs that are biomimetic to the entire nervous system. The emulation of both neurodegenerative disorders and neural stem cell behavior patterns in micro-platforms is also provided, which can be used as a basis to construct nervous system-on-a-chip.

## 1. Introduction

Neurodegenerative diseases, the loss of the function of neurons and their resulting cell death, are one of the serious diseases that threaten one’s quality of life as it progresses [1,2]. In the case of Alzheimer’s disease, one of the representative neurodegenerative disorders, approximately 5.4 million Americans were reported to be affected in 2016, and eleven percent of Americans over 65 suffer from it [3]. Though these neurodegenerative diseases are affected by age, there are no significant therapeutic methods yet, because their causes are directly related to neuronal cell death, whose revival and functional recovery are very difficult [4,5]. In addition, early diagnosis for preventing and delaying the progress of disease is challenging, due to the signature biomarkers being blocked by the blood–brain barrier (BBB) [6,7]. The BBB, which consists of brain microvascular endothelium and its tight junction, keeps the brain safe from pathogens and other toxins [8,9,10]. While it protects the central nervous system (CNS) from the virulent factors, it also inhibits translocation of drugs from the blood vessel to neuronal tissue. Therefore, the BBB presents as one of the critical barriers to both the diagnosis and therapy of neurodegenerative diseases. Furthermore, the complexity of the brain and its intricate cellular network, which consists of astrocytes, neurons, oligodendrocytes, a vascular endothelium, pericytes, and immune cells such as microglia and lymphocytes, adds major challenges in developing successful treatments. 

There have been several in vitro model systems developed to study the pathophysiology and therapeutic strategies of neurodegeneration [11,12,13]. However, compared to the human nervous system, conventional cell culture systems, including co-culture in Transwell systems, have some limitations, such as the difficulty of maintaining stable oxygen and nutrient concentrations, as well as limited application of shear stress, which can affect inflammatory responses through reduced cytokine stimulation and activation of the endothelium, which induces the transmigration of leukocytes. On the other hand, animal models have been considered as an alternative to studying the human nervous system, due to their relatively similar structures when compared in vitro. However, animal models present their own sets of challenges, considering the discrepancy in physiological responses compared to humans. Also, their intricate structures provide too many variables for the specific causal relationship analysis of pathophysiology and therapeutic effect [14]. 

Recently, scores of researchers have placed a greater emphasis on studies of in vitro human brain models utilizing micro-platforms, such as microfluidic channels and three-dimensional (3D) human cell culture systems. Nervous system-on-a-chip, consisting of designed micro-scale polymeric channels for the manipulation of nano liter sample volumes and several types of human neuronal cells, focuses on the reconstitution of 3D microstructure and tissue–tissue interface of both human CNS and peripheral nervous systems (PNS) [15,16]. While maintaining the advantages of in vitro models, such as utilizing human cells, a micro-platform-based cell culture model can provide the cells with more physiologically relevant conditions, such as controllable fluid flow, shear stress, and mechanical deformations. Using this platform, it would be possible to emulate organ-level functions relating to homeostasis, or pathophysiological responses with molecular resolutions [17,18]. In addition, the microfluidic system has the potential to revolutionize the ability to perform procedures, such as pathophysiological approach and high-throughput drug screening against diseases, including neurodegenerative disorders, as well as non-invasive in situ biological sensing [19,20,21,22]. 

In this review, we present in vitro micro-platform-based neuronal cell maintenance and analysis systems for the emulation of in vivo human microphysiological events in the CNS and PNS. Due to the complexity of the different interconnected cells of the nervous system, the reconstruction of the neuronal network between cells is one of the significant topics highlighted in literature: such a system mimics the human neuronal system using two-dimensional (2D) or 3D microstructure with appropriate microenvironmental factors. On the other hand, neuronal cells in the actual human nervous system can be influenced by the microvascular endothelium and its secretomes, as well as the leukocytes from blood vessels. These kinds of interactions can be reconstituted in microfluidic chips that utilize co-culturing techniques while applying shear stress comparable to that of the body-like fluid. Two representative nervous system-on-a-chip technologies, demonstrated by analytical technologies such as neurotransmitter detection and calcium staining, that can evaluate the therapeutic effect against neurodegenerative diseases or determine disease progression, are subsequently described.

## 2. In Vivo Mimicking Central Nervous System Model on a Microdevice

The CNS, composed of the brain and the spinal cord, is based on the connections between various types of neural cells called neural networks. Understanding the physiological behaviors of neurons and their working mechanisms in the CNS is essential for finding the underlying causes of brain diseases such as neurodegenerative diseases. Due to the complexity of the CNS and limited access to humans as experimental subjects, the development of an in vitro platform capable of mimicking the in vivo neural environment is required. This section will discuss how the current in vitro two-/three-dimensional neural network can be fabricated to mimic in vivo human neural networks as one organ, neurodegeneration, and regeneration model. The advantages and limitations of these methodologies are summarized in Table 1.

### 2.1. Two-Dimensional Neural Network on a Microdevice

**Figure 1:**
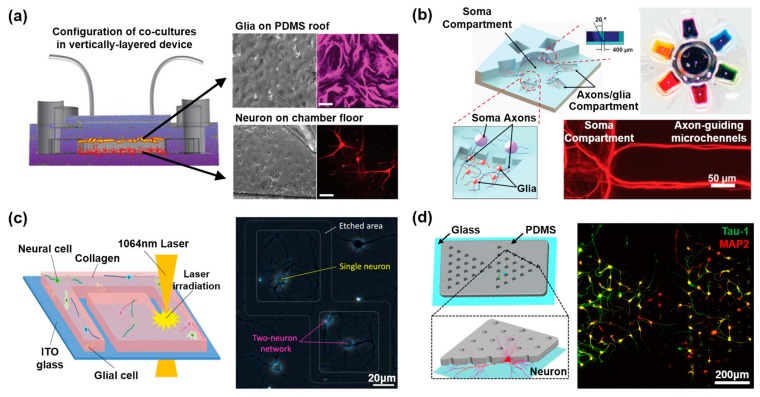
2D and 3D neural networks on the microdevice. (**a**) Co-cultures of neurons and glia in the vertically-layered configuration. A monolayer of glial cells (polydimethylsiloxane (PDMS) roof) was immunostained with the glial-specific marker glial fibrillary acidic protein (GFAP). mCherry transfected neurons (chamber floor) were viewed in fluorescence. Reproduced with permission [30]. Copyright 2013, Royal Society of Chemistry; (**b**) 3D illustration and images of the multi-compartment neuron–glia co-culture microsystem capable of carrying out multiple localized axon treatments in parallel axons from neuronal soma, for localized axon–glia interaction studies. Reproduced with permission [26]. Copyright 2012, Royal Society of Chemistry; (**c**) Control of culturing area and the number of neurons using collagen gel photothermal etching to monitor the synaptogenesis. Reproduced with permission [28]. Copyright 2013, Royal Society of Chemistry; (**d**) The uniformly aligned neural network was generated with the patterned hole array platform for interrogating neural circuitry. Reproduced with permission [29]. Copyright 2014, Nature Publishing Group.

In the CNS, it is well known that there are several neurons and glial subtypes that need to be cultured together so that they may communicate together to build a supportive extracellular matrix (ECM) [48]. To mimic the microenvironment of the CNS on the microdevice, various ECM materials have been utilized, including fibronectin, polylysine, laminin, albumin, and peptides for the control of cell adhesion and growth [49]. With the ECM-coated microdevice, many researchers have utilized several stem cell types, including neural stem cells (NSCs), embryonic stem cells (ESCs), and induced pluripotent stem cells (iPSCs), to mimic in vivo neural networks by inducing differentiation [50,51,52]. After the differentiation of stem cell into the neural lineage, a mixed population of differentiated neural cells (such as neurons, astrocytes, and oligodendrocytes) are obtained and co-cultured in a culture plate [53,54,55]. Conventional tissue culture plate based co-culture methods are limited for neuronal network research, considering that neurons extend their neurites over a long distance, randomly establishing connections with other types of neural cells [23,24]. In addition, understanding the interactions of each cell component is limited, in that there are several types of interactions, including neuron–astrocyte, neuron–oligodendrocyte, and neuron–neuron, with overlapping cellular responses. To this end, micropatterning and microfluidic technology enables the fabrication of multi-chamber cell culture platforms that allow for the compartmentalized culturing of different cell types, as well as the guidance of different cell growths to connect to each other. Shi et al. developed a vertically structured microfluidic chip, composed of four chambers for the neuron–glial co-culture [30] (Figure 1a). In the vertically-layered configuration, glia and neurons were co-cultured with a monolayer of glial cells on the polydimethylsiloxane (PDMS) roof of the cell chambers, while neurons were cultured on poly-l-lysine (PLL)-coated bottom surfaces of the cell chambers within the same device. By using the vertically compartmentalized device, this microfluidic system was able to show the secreted cytokine based communication between glia and neurons. In particular, the glial cells have improved the formation and stability of the synaptic contacts between neurons. However, unlike the in vivo nervous system, the vertically-layered device is not able to represent the physical interaction between each cell type, such as paranodal region formation [25]. As a different approach to neuronal–glial interaction, Park et al. developed a multi-compartment neuron–glial co-culture microsystem capable of carrying out multiple localized axon treatments in parallel [26] (Figure 1b). It is shown that astrocytes were found to physically damage the established axonal layer, as they tend to grow underneath the axons and induce weaker interactions between axons and substrate, while oligodendrocytes align to neighboring axons. Since the soma and axons/glia compartments of the device were connected through microchannel, secreted cytokines or treated chemical in a specific section of the device, can diffuse into other sections. As an alternative design to a solid-PDMS based microchannel or wall, a microfluidics based oil barrier system was reported, which can connect/block the separated chambers for the co-culture platform [27]. Similarly, various methods have also been reported to mimic and understand the neuron to neuron interactions. Odawara et al. established an in situ photothermal collagen gel etching method for the generation of spatially controlled neuronal network patterns on chip platforms [28] (Figure 1c). The synaptogenesis of these constructed neural networks, containing specified numbers and types of cells, was accurately monitored, while controlling the direction of neurite elongation. Despite the strong advantage of the photothermal neuronal network guiding method, it is limited to control the large area. By fabricating a micro-hole array platform, seeded neurons are growing and integrating the differentiated neuronal circuit. [29] (Figure 1d). This array platform allowed for the neuronal signal transmission and distribution patterns to be monitored.

Consequently, through the fabrication of a microdevice with a compartmentally controlled 2D neural network, understanding the interaction between neural cell components and their behavior in CNS is possible. The fabricated 2D neural network microdevice also can be utilized as a drug screening platform, which enables the monitoring of each or both cell composites’ cellular responses. While the 2D neural network system has aforementioned advantages, it still has, nevertheless, limitations in mimicking the 3D tissue specific characteristics, such as nutrient diffusion and 3D axonal growth, because of the absence of surrounding scaffolds which can support 3D cell growth [31,32,33].

### 2.2. Three-Dimensional Neural Network on a Microdevice

**Figure 2:**
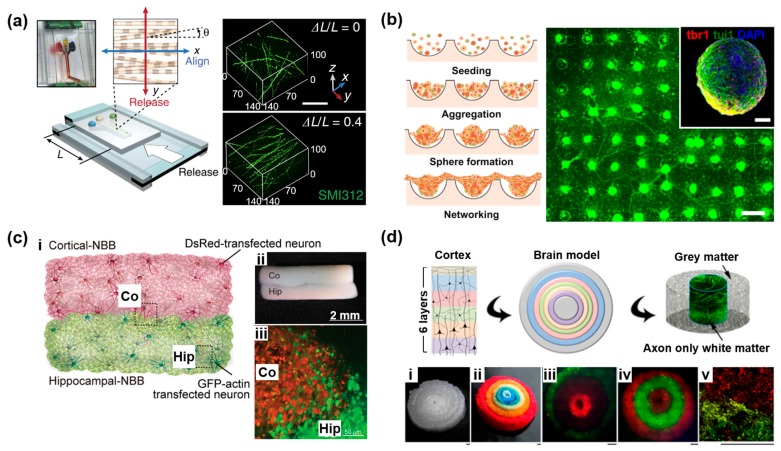
3D neural network on the microdevice. (**a**) Schematic diagram and confocal microscopy image of reconstruction of an anisotropically organized hippocampal neural network. Reproduced with permission [37]. Copyright 2017, Nature Publishing Group; (**b**) Formation of a networked neurosphere model in the PDMS micro-concave wells. Reproduced with permission [56]. Copyright 2013, Elsevier; (**c**) Neural pathway formation by the assembly of different types of neural building blocks (NBBs). (**i**) Schematic illustration of axonal extensions between NBBs. (**ii**) Stereomicroscopic image of NBB assembly of cortical-NBB (Co) and hippocampal-NBB (Hip). (**iii**) Fluorescence image of an assembled NBB, using Cell Tracker green labeled cortical-NBB and Cell Tracker red labeled-hippocampal-NBB. Reproduced with permission [57]. Copyright 2012, Royal Society of Chemistry; (**d**) 3D assembled cortex mimicked tissue structures. (**i,ii**) Six layered donuts of silk scaffold with original (**i**) and dyed color (**ii**), (**iii**–**v**) two three-layered cortical neuron constructs, (**v**) neurons at the interface. (Scale bar: 1 mm). Reproduced with permission [39]. Copyright 2013, National Academy of Sciences of the United States of America.

Unlike the conventional 2D in vitro cell culture systems, in vivo tissue is comprised of 3D constructs. The brain is particularly composed of anatomically distinct elements interconnected by 3D neural networks. Hydrogels are popular materials, capable of supporting the growth of suspended adherent cells for 3D neural network formation. The native structure of neural tissue can be mimicked by hydrogels using natural or synthetic polymers, including ECM materials such as hydrogel components [31,32,33]. For example, hydrogels successfully assisted the 3D neural network formation from ESC spheroids [34,35]. By encapsulating the spheroid with hydrogels, cells are supported on a 3D architecture and able to grow in every direction. In particular, Lancaster et al. showed that collagen based hydrogels can mimic the structural environment of the brain development process [34]. Differentiated cells from the encapsulated ESC spheroid in the hydrogel formed a laminar structure by themselves, without any external stimulation or structural assistance. However, cells were not fully aligned like actual brain tissues in each layer of the laminar structure. Similarly, Bae et al. showed the ESC derived interconnected neurosphere array as a 3D neural network, by using synthetic polymer based hydrogels [35]. The ESC embedded hydrogel hemisphere array was fabricated using the concave microwell array as a mold. After neurodifferentiation, ESCs differentiated into neurons and formed the neurosphere inside of the hemisphere. When the axons of differentiated neurons reached the boundary of the hydrogel hemisphere, they started to stretch randomly outward. Although spheroid embedded systems have been shown to successfully generate neural networks, it should be noted that neural networks do not fully mimic brain tissue, because neurite outgrowth occurs randomly in all directions. Further, these systems limit compartmental control to study cell-specific features [38]. To address this drawback, researchers have developed a multi-layered hydrogel system embedded with neural cells, constructed to mimic the specific structure of brain tissues [36,37]. Kunze et al. on the other hand, developed a multilayered agarose–alginate scaffold that mimics the layered organization of the neocortex in a microfluidic chip [36]. Nonetheless, most of the neurons in the brain projected their axons vertically/horizontally into other regions in the brain with columnar structure. Since those columnar structures were organized with the long developmental process, it was hard to control in the hydrogel based culture condition. By taking advantage of microfluidics, Kim et al. showed the reconstruction of a 3D hippocampal neural network in a monolithic gel, in which CA3 neurons extended parallel axons that synapsed with CA1 neurons [37] (Figure 2a). Through this technique, these researchers provide compartmentalized neuronal growth to mimic the linear architecture of brain, such as in the cerebral cortex.

Although hydrogels have been used as scaffolds for 3D cell cultures in many applications, the cell embedded hydrogel culture systems have some limitations of their own, including limited nutrient transportation and reduced cell viability [58]. In addition, hydrogel cultures suffer from contraction or loss of integrity over time, which limits culture times and function [38]. To address these limitations, gel-free spheroid formation methods have been developed as a bottom-up approach to tissue engineering [56,57,59,60,61,62,63]. For instance, Choi et al. have shown the formation of cortex mimicking neural spheroids and their networks, using neural progenitor cells in arrayed concave microwells with hydrophobic surfaces [56] (Figure 2b). Furthermore, the individually constructed neurospheres can be utilized as a neural building block. Kato-Negishi et al. generated two different neural building blocks that replicate the modular interaction between the cortex and hippocampus [57] (Figure 2c). These techniques enable not only visualization of the spatiotemporal morphological changes of single neurons during axonal extension and synaptic formation, but also mimicking the interaction of neurons at the interface between the different region of the brain. The microfabricated neurospheroids are formed solely out of neurons, however, and they are thus limited in size to 150 μm, because of the limits of oxygen diffusion. 

One of the alternative methods to overcome the limitation of hydrogel and spheroid based nervous system mimicking is the utilization of sponge-like biomaterial scaffold to form the 3D neural network. Numerous scaffolds have been produced from a variety of biocompatible materials, such as synthetic polymers (poly-l-lactic acid (PLLA), polyglycolic acid (PGA) and poly-lactic-co-glycolic acid (PLGA)) and natural polymers (collagen, various proteoglycans, alginate-based substrates, and chitosan) [64,65,66,67,68,69,70]. For example, Tang-Schomer et al. developed a silk–collagen composite scaffold that epitomizes the compartmentalized nature of the cortex [39] (Figure 2d). The use of silk showed the mechanical stability and ease of handling of the 3D brain tissues, which cannot be achieved with methods based solely on soft hydrogels. The stable silk sponges also serve as anchoring support for the central collagen gel, to avoid loss of volume or shape over time while in tissue culture [38]. 

To summarize, the 3D neural network on the microdevice has several characteristics that make it an attractive alternative for 3D neural tissue models. By using different biomaterials and culture methods, constructed 3D neural network models showed more similar properties as neural tissues, however, they still have some nutrient diffusion related limitations, such as necrosis and hypoxia. Adding the neurovascular structure on the neural network system is one of the possible breakthroughs for the 3D neural network model. 

### 2.3. In Vitro Neurovascular Unit Models in the Microfluidic Device

The entire nervous system, including the brain, has an insufficient capacity of glucose and oxygen storage, and depends greatly on blood vessel supplements, such as oxygen and nutrients. In addition, the functional dependence and communication between the nervous and vascular systems, referred to as neurovascular coupling, are demonstrated in several publications [71,72,73]. For example, the control of cerebrovascular functions at different levels of the vascular tree is essential for the regulation of cerebral blood flow and for the confirmation of metabolic requirements of neurons inside the brain [74,75,76]. Therefore, it is essential to construct the integrated structure of the nervous system from microvascular constituents, including brain microvascular endothelium, tight junctions, gap junctions, and blood flow, like shear stress, to emulate the in vivo human nervous system. For the accurate mimicry of the neurovascular unit, which is composed of blood vessels, the BBB, and neuronal cells, microfluidic systems can be a prominent platform technology. In this section, we describe how the current microfluidic techniques can be integrated for the replication of the in vivo human neurovascular unit as a one organ system, for the induction of neurodegeneration and regeneration.

**Figure 3:**
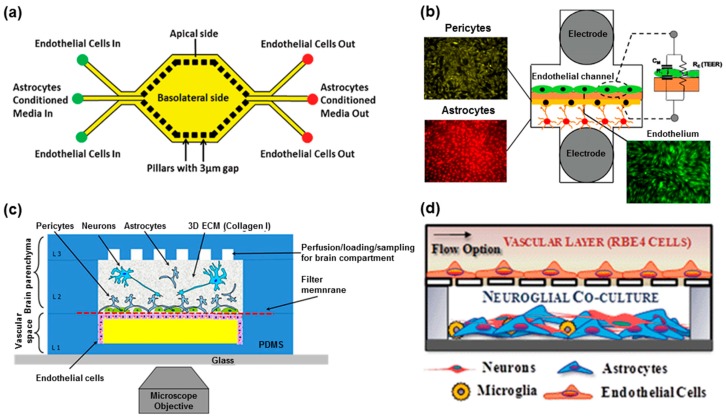
Reconstitution of an in vitro neurovascular unit using the microfluidic platform. (**a**) Microfluidics-based Synthetic Microvasculature model of the Blood–Brain Barrier (SyM-BBB). Apical side consists of endothelial cells, while basolateral side contains astrocytes conditioned media. Reproduced with permission [87]. Copyright 2013, Royal Society of Chemistry; (**b**) The design of the layered microfluidic channels and the equivalent circuit model. Electrodes are embedded on opposing sides bEnd.3 cells and pericyte cultured on a polyester porous membrane. Reproduced with permission [42]. Copyright 2016, American Chemical Society; (**c**) In vitro microfluidic neurovascular unit (NVU) indicating endothelial cells lining the lower, vascular chamber; astrocytes and pericytes lining the other side of the filter membrane, with neurons in the collagen gel in the upper brain chamber. Reproduced with permission [43]. Copyright 2015, American Institute of Physics; (**d**) The neurovascular microdevice was assembled with both the vascular layer with a flow option and the neural chamber with 3 different neuronal cells. Reproduced with permission [44]. Copyright 2013, Royal Society of Chemistry.

As mentioned previously, the BBB is a unique structure in the neurovascular system that prevents unwanted materials from entering, while allowing the passage of small molecules and certain biomaterials. Many in vitro BBB models using an endothelium with tight junctions and gap junctions have been developed to provide a platform for screening the efficacy and toxicity of therapeutics to the CNS [77,78,79,80]. To construct a BBB model, there should be two spaces separated by the vascular endothelium. The Transwell system, which consists of smaller wells with micro-sized holes inserted into an outer well, could satisfy such a condition [81]. Many researchers have utilized it as co-culture system for the vascular endothelium, astrocytes, pericytes, and neurons; however, its emulation of the neurovascular unit is flawed due to its inability to apply a shear stress (such as blood flow) on the vascular side [82,83,84,85]. Several studies have successfully demonstrated the mimicry of human BBB structures using microfluidic devices that can replicate blood flow. Yeon et al. developed a microfluidic device for mimicking the BBB and testing CNS drug permeability, by trapping human umbilical vein endothelial cells (HUVEC) on micro-holes with a medium flow [86]. These trapped HUVEC cells are in tight contact with each other, constructing a tight junction-like structure that absorbs the drugs. The effect of hydrogen peroxide, mainly via the induction of reactive oxide species (ROS), on the transendothelial permeability of the BBB has also been shown. Hydrogen peroxide can induce disruption of the tight junction, and increase of the transendothelial permeability as a result. It was successfully exhibited to increase the permeability by hydrogen peroxidase within a short period of time (below 1 h). However, this system did not emulate the shear stress and flow direction of the blood vessel, and did not use any neuronal cells. Prabhakarpandian [87] et al. have demonstrated a hexagonal structured microfluidic chip with two separate inlets and outlets, one for the endothelium, and the other for the astrocyte cell culture [87] (Figure 3a). Inside the hexagonal shape, astrocytes comprise the basolateral side with a 3 µm gap pillar connected to the outer channel where the endothelium is attached. The P-glycoprotein (P-gp) and tight junction protein (ZO-1 and Claudin-1) were upregulated significantly under flow in a microfluidic model with an astrocyte-conditioned medium, compared to the Transwell system. This microfluidic system can also regulate the transporting efficiencies of the endothelium, indicating a functional representation of the in vivo BBB, by upregulating P-gp and efflux transporters. However, its structure was quite different to the neurovascular unit; it did not present neurons and pericytes on the basolateral side of the microfluidic chip, and the astrocyte was attached on the basal plate, and not on the 3D ECM structures. Kim et al. presented a 3D brain microvasculature system embedded within the bulk of a collagen matrix, using microneedles and a 3D printed frame [88]. This model allowed for the demonstration of a time-dependent evolution of the barrier function for up to 3 weeks, and showed successful disruption and recovery of the barrier function upon application of a hyperosmotic mannitol solution. Even though its vascular structure was quite similar, a similar permeability value could not be shown, due to nothing being attached to neuronal cells on the opposite side of the vascular endothelium. As such, there are several attempts to emulate and recapitulate the complex neurovascular unit by recreating the vascular–neuronal tissue interface and its interaction with a microfluidic device [86,87,88]. But, there is also lots of room to improve for close mimicking of the structures and physiological responses, such as 3D ECM structures with astrocytes and other neuronal cells with similar oxygen and nutrient concentrations.

Similarly, there have been vertically aligned multichannel microfluidic chips for the mimicry of neurovascular structures with similar and intuitive assembly [40,41,42,43,44,85]. These microfluidic chips have a vertically ordered co-culture system with a porous membrane between cell layers, designed to induce polarization of the vascular endothelium, to create an apical and basolateral side. This design also promotes cell–cell interaction between astrocytes and pericytes on the basolateral side, and can initiate secretome release for intercellular communication [45,46]. One of the studies described a BBB, consisting of an endothelium and astrocytes attached on opposing sides of a porous membrane, effectively emulating a dynamic cerebrovascular environment with the fluidic flow [40]. To monitor the trans-endothelial electrical resistance (TEER) values, which is the key readout to measure BBB integrity, two electrodes were inserted in the top and bottom channel. The TEER values were found to be significantly higher than those in static models. Also, a transient drop and recovery of the TEER were observed by applying histamine, indicating the robustness of the model for repeated long-term testing purposes. In another case, it was shown that co-culturing astrocytes with a vascular endothelium can promote leukocyte transmigration, as well as an increase in BBB integrity, upon application of an appropriate shear force using a 3D flow chamber [41]. In this research, they demonstrated that abluminal astrocyte processes protruded through membrane pores and contact luminal endothelium, a similar structure to in vivo neurovascular units. Based on the results, they claimed that this model offers the opportunity to evaluate BBB properties and leukocyte transmigration across cytokine-activated vascular endothelium as influenced by human astrocytes. Wang et al. also developed a triculture microfluidic 3D BBB model composed of vascular endothelium, pericytes, and astrocytes [42] (Figure 3b). This model demonstrated high TEER values with low permeability for [14C]-mannitol and [14C]-urea on the triculture layer, and exhibited high functional expression of the P-gp efflux pumps. However, the aforementioned microfluidic devices do not sufficiently mimic the natural 3D in vivo microenvironment, due to the lack of essential ECM materials, such as collagen, fibronectin, and laminin, all of which are crucial components of the neurovascular unit. Cell recognition of ECM via cell surface receptors can trigger several cellular responses such as secretion, proliferation, migration, and differentiation, underlying the significance of ECM for mimicking the human nervous system [47,89]. To mimic the actual human neurovascular structure, some of the models have neurons and astrocytes, as well as other components, including microglia or 3D ECM structures. For instance, in such a construction, neuronal cells, including neurons and astrocytes, are maintained in an ECM like scaffold, while pericytes and vascular endothelial cells are attached to the porous membrane that is present between the channels [43] (Figure 3c). The BBB integrity in this microfluidic system had been validated with both fluorescein isothiocyanate (FITC)–dextran diffusion and TEER values. They tested the disruption of the BBB of this system by exposing it to 1 mM glutamate, and significantly increased diffusion of FITC–dextran across the BBB was shown. Achyuta et al. showed that the endothelial barriers prevent the translocation of dextran through neurons, astrocytes, and microglia [44] (Figure 3d). In addition, it was successfully demonstrated that upon the addition of an inflammatory agent (tumor necrosis factor alpha, TNF-α) into the vascular channel, the activation of microglia and astrocytes was observed through the upregulation of intercellular adhesion molecule 1 (ICAM-1), a characteristic receptor for leukocytes, on the surface of the vascular endothelium. But, there is also a limitation to emulate the actual neurovascular unit. One of the limitations is the use of PDMS to create the channels and membrane, given the property of hydrophobic molecules to adsorb to the PDMS, that has also the hydrophobicity to generate hydrophobic interactions with non-polar small molecules, such as the hydrophobic tails of the lipid and the hydrophobic residues in proteins [90].

To sum up, microfluidic systems make it possible to reconstitute the neurovascular unit, in vitro, with appropriated medium supply and shear stress, however, they should be improved in terms of the co-culturing, migration, and activation of immune cells, and incorporation of 3D ECM for the closer development of human neurovascular function.

## 3. Neuronal Disease Models on the Nervous System-On-A-Chip

Neuronal diseases, such as Alzheimer’s disease and glioblastoma, are critical disorders that severely affect patients’ quality of life, since no effective cure exists. Furthermore, developing appropriate therapeutic methods and drugs is difficult, due to the constraints pertaining to studying the BBB. There are several reasons that make treating neuronal diseases difficult, including the physiological and structural complexity of the brain, and the poor transport of the drug across the BBB. Therefore, it is necessary to develop in vitro human neuronal models as drug-screening platforms, as well as investigative tools for the study of cause and progression. Microfluidic technology can better reconstruct the in vitro neuronal disease states by emulating the neurovascular unit. This allows for the fine-tuning of a wide range of parameters, including flow rate and cell–cell interaction, while using small volume amounts, such as few nanoliters, which is advantageous for the analysis of biological events. In this section, it is demonstrated that the phenomenon of neuronal diseases has been successfully replicated in the microfluidic device with human neuronal cells. The advantages and limitations of these methodologies are summarized in Table 2.

### 3.1. Neurodegenerative Disease Models on the Nervous System-On-A-Chip

**Figure 4:**
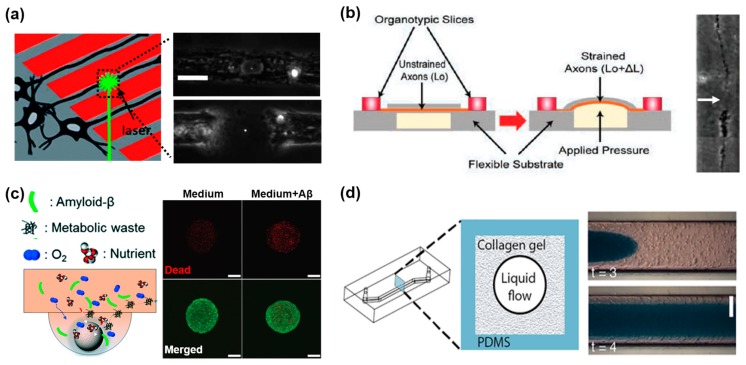
In vitro microfluidic devices for mimicry the neurodegeneration and neural stem cell (NSC) differentiation. (**a**) Axonal injury model by laser induced axotomy to provide precise damage to selected neurons. Reproduced with permission [92]. Copyright 2010, Royal Society of Chemistry; (**b**) Diffused axonal injury model in a microdevice with a flexible substrate, which can apply pressure to damage neuron. Arrow indicates the axonal degeneration 20 h post injury. Reproduced with permission [93]. Copyright 2014, World Scientific; (**c**) Alzheimer’s disease brain mimicking microfluidic chip, which consists of neurospheroids, are cultured under normal medium containing oxygen, nutrient, and 5 μM synthetic amyloid-β (1–42). Reproduced with permission [62]. Copyright 2015, Royal Society of Chemistry; (**d**) The pressure-driven microfluidic model to generate the cylindrical collagen gel channel in the 3D BBB chip. A continuous hollow cylindrical lumen channel was utilized as the microvasculature with a vascular endothelium. Reproduced with permission [96]. Copyright 2016, Herland et al.

Neurodegenerative diseases are characterized by the progressive loss of structure or function in neurons, leading to neuronal death. The simplest neuronal damage to mimic in a neurodegenerative disease model is axonal injury, which is a disconnection of the axon and resultant loss of the signal through the neuronal system. A decade ago, a novel microfluidic culture platform was utilized for the polarization of CNS axon growth [91]. The developed microfluidic culture system could polarize the growth of axons into a chamber, facilitating biochemical analyses of pure axonal fractions and localizing physical and chemical treatments to axons or soma. It was also validated as a potential method to screen candidate molecules for axonal regeneration. The authors showed that the isolated axons from the cell body in neurotrophin-treated chambers, such as brain-derived neurotrophic factor (BDNF) and neurotrophin-3 (NT3), had a dramatic increase in axonal growth. Specifically, axons were co-cultured with oligodendrocyte, to see the myelination and confirm its success. In the other study, a micropatterned substrate was utilized to induce aligned axonal growth, while a pulsed laser microbeam was used to cut the axon from the soma [92] (Figure 4a). This method allowed for the study of the dynamics of axonal injury and regrowth under controlled conditions, by adjusting laser microbeam pulse energy and applying ethylene glycol-bis(β-aminoethyl ether)-*N*,*N*,*N*′,*N*′-tetraacetic acid (EGTA) as a model drug. EGTA works by chelating extracellular calcium, to alleviate degenerative changes. Using laser microbeam dissection within a micropatterned substrate could produce precise zones of neuronal injury, and shows te potential for high-throughput screening of agents to promote neuronal regeneration though laser-associated damage is quite different from the actual trauma or spinal cord injury. Also, a traumatic brain injury (TBI) model was developed on a similar microdevice, which can apply uniaxial pressure to make strained axons that consequently emulate a diffused axonal injury [93] (Figure 4b). It is suggested that the axonal diameter plays a significant role in strain injury, which mimics the cause of TBI. As the axonal diameter increases, the number of axonal beading decreases. These microdevices can be used both to understand the axonal degeneration, and screen for potential therapeutic agents. The axonal degeneration model could provide an easy and simple platform to study neuronal damage against toxic materials, and physical shock and its effect, as well as the drug screening platform [91,92,93]. However, to mimic the actual damaged nervous system, the aforementioned microdevices missed the essential component: the interaction between neurons and glial cells. For example, by implementing oligodendrocytes in the system, myelination and demyelination of neurons can be achieved [48,101,102,103].

Besides the axonal injury model, neurodegenerative disease models have been reproduced in microfluidic devices to study their pathophysiology and utilize as a drug screening tool [62,94]. Among them, Alzheimer’s disease (AD) is one of the most studied and established models, due to the high maintenance cost on the afflicted. Most AD models utilize amyloid-beta (Aβ) to induce the disease and neuronal death. Cho et al. developed a microfluidic chemotaxis platform to study microglial accumulation in response to week-long gradients of soluble Aβ and patterns of surface-bound Aβ [94]. This platform is composed of a large central reservoir applying Aβ, and two side reservoirs containing a medium. Human microglia were loaded in the annular compartment, and their migration was observed towards the central compartment. They found that soluble Aβ provides recruiting signals, while surface-bound Aβ acts as a targeting signal for the induction of microglia migration towards the Aβ concentrated region. Using this platform, it was uncovered that Aβ can induce the microglial migration easily in a time-dependent manner through microglia maintained in the 2D culture substrate. However, it was not able to mimic the whole AD progress and functional change, even though microglia migration is one of the critical phenomenon leading AD progress. Park et al. established a microfluidic 3D neurosphere model with a low fluidic flow rate to mimic the interstitial flow in the brain [62] (Figure 4c). In this system, the 3D spheroid culture emulates the cell–cell interactions found in the brain, while the flow helps deliver nutrients and oxygen, and eliminates the metabolic wastes like an interstitial flow within the brain. Also, the toxic effects of Aβ were tested via an osmotic micropump, confirming a reduction in the viability of the neurospheres, and significant destruction of the neural networks as a result. Comparing the 2D culture system, neurospheres were more reliable to analyze the effect of the Aβ accumulation, and they showed spheroids have much more viability than that of 2D culture. However, there was also a limitation, because it could not emulate the intercellular interaction between neuronal cells and vascular cells related to the accumulation and elimination of the Aβ. 

### 3.2. Neuroinflammation Models on the Nervous System-On-A-Chip

In neurodegenerative diseases, neuroinflammation is one of the main mechanisms for the induction of neuronal loss in the nervous system, through the secretion of pro-inflammatory cytokines from astrocytes and microglia [104,105]. In this pathophysiological process, there are several cells that participated directly or indirectly and interaction between cells, such as vascular endothelium and monocyte, that are necessary to generate an inflammatory response. Therefore, it is essential to study the neurovascular unit as one organ system for mimicry of neuroinflammation and neurodegenerative disease. In vitro neurovascular models have been fabricated to induce a neuroinflammatory state to validate the functionality of the model. One study developed a 3D BBB model using brain endothelium surrounded by poly (d-lysine) and collagen type I [95]. The induction of the neuroinflammatory state utilized TNF-α, one of the representative pro-inflammatory cytokines, and ischemia by oxygen–glucose deprivation with a neutrophil injection. It was shown that the BBB mimic in this model was disrupted upon exposure to TNF-α and ischemia. But, there were some limitations: not using astrocyte or other neuronal cells, and applying TNF-α to induce neuroinflammation. TNF-α is one of the secretomes from cells, and not toxic materials, or an induced substance of neuroinflammation. Herland et al. demonstrated how astrocytes and pericytes contributed to the neuroinflammatory phenomenon when they applied TNF-α in an engineered microvessel [96] (Figure 4d). In order to develop the BBB model in vitro, it is important to mimic these key physical features of the brain capillary microenvironment, including fluid flow, 3D ECM mechanics, and the cylindrical geometry of normal brain microvessels. The cylindrical collagen gel was formed applying hydrostatically-controlled medium flow, to make a liquid flow channel in the filling the channel with a solution of type I collagen and astrocytes and/or pericytes. After making the chip, they seeded the vascular endothelium into the channel to make a neurovascular unit. It was shown that the secretion of granulocyte colony-stimulating factor (G-CSF) and interleukin 6 (IL-6) depend on the presence of astrocytes or pericytes, and was significantly increased when compared to that of Transwell co-cultures. They mentioned that the major difference between this microfluidic chip and Transwell cultures is that these other models contain semi-permeable membranes that separate the interacting cell types, whereas compliant ECM gels are constrained within a confined cylindrical geometry, as well as apply shear stress on the endothelium layer. It could be improved by adding essential immune cells to mimic the neuroinflammation, such as microglia and monocytes from the vascular channel. To better understand the detailed metabolism of neuroinflammation and BBB disruption, one group developed a dual-chamber neurovascular unit-on-a-chip, and applied lipopolysaccharide (LPS), which is one of the main inducers of the neuroinflammation, along with a cytokine cocktail of interleukin 1β (IL-1β), TNF-α, monocyte chemoattractant protein (MCP)-1, and MCP-2, to analyze the BBB response via cytokine detection and mass spectroscopy analysis [97]. The study concluded that this system was able to emulate the initial effects of neuroinflammation and pro-inflammatory cytokine activation upon the disruption of the BBB. On the contrary, metabolic pathway changes induced the recovery of the BBB during exposure to cytokines. In this way, there were diverse trials to emulate the neuroinflammation by microfluidic technology, however, most focused on the endothelium and vascular interaction, not the neuronal damage and functional loss by immune reaction with immune cells. 

### 3.3. Metastatic Brain Tumor Model on the Nervous System-On-A-Chip

**Figure 5:**
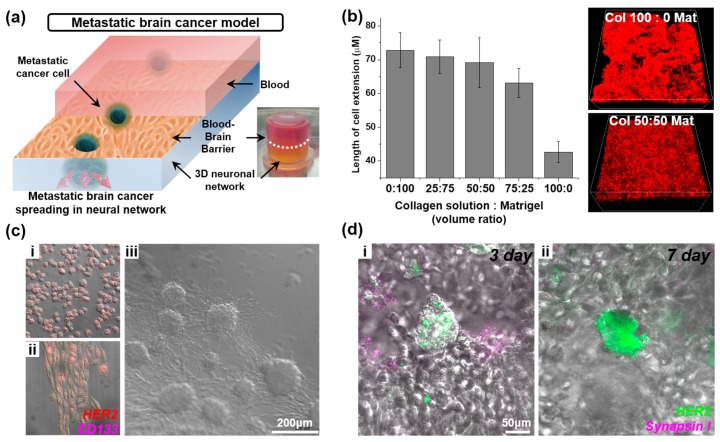
Metastatic brain tumor model on the nervous system-on-a-chip. (**a**) Schematic illustration and image of metastatic brain cancer model system; (**b**) 3D neural cell network in a different ratio of collagen I solution and Matrigel mixture. Neurite outgrowth was affected by the concentration of collagen in the hydrogel; (**c**) Morphological differences (**i**) before and (**ii**) after differentiation of cancer stem cells (CSCs). (**iii**) CSCs spreading in hydrogel block (50:50 mixture of collagen I and Matrigel). (HER2: red, magenta: CD133) (**d**) Fluorescence image of breast CSC cell cluster in the 3D neural cell network, three and seven days after the seeding. (HER2: green, magenta: Synapsin I).

CNS involvement typically occurs late in the course of metastatic cancer [106]. To understand the metastatic process and to know the effect of cancer on the brain, a model that mimics the neural network is essential [107]. For this purpose, several hydrogel-based cancer spreading models mechanistically mimicking brain tissue stiffness have been reported [98,99,100]. However, due to the lack of neural cells being contained in these hydrogel systems, little is known about the interactions between metastatic cancer cells and the surrounding neural networks. To this end, we have fabricated 3D neural networks in a customized hydrogel system to monitor the effect of metastatic cancer cells on neural networks (Figure 5a).

The immortalized neuroblastoma cell line, SH-SY5Y, is one of the most commonly used cell lines for neuroscience research [108]. Since differentiated SH-SY5Y expresses neuronal markers, such as neurofilaments and microtubule-associated protein 2 (MAP2), it was utilized to construct the 3D neural cell network in this work [109]. To obtain an optimized condition for the formation of a neural cell network, two types of hydrogel were mixed together with varying ratios of collagen type I (3.0 mg/mL) and Matrigel. Although Matrigel is one of the most popular hydrogels for biomedical applications, such as monitoring cell migration and formation of 3D cell structures, its stiffness is incompatible with the structure of ECM in brain tissue [110]. Collagen type I was mixed with Matrigel to overcome this problem, by increasing the elastic modulus of the hydrogel. A mixed volume ratio (0:100, 25:75, 50:50, 75:25, 100:0 of collagen solution and Matrigel) was tested on SH-SY5Y (10^6^ cells/100 μL) by monitoring neurite outgrowth (Figure 5b). The collagen-only hydrogels yielded neural cells with short neurites, when compared to the hydrogels composed of mixed collagen. However, as the collagen ratio decreased, neural cell morphology was stretched. A hydrogel ratio of 50:50 was found to yield an ideal condition, showing homogenous cell networks with well-differentiated neuronal morphologies. With this in mind, specific breast cancer stem cells’ (CSCs) metastatic characteristics, including differentiation and proliferation traits, were studied prior to the fabrication of a brain metastasis model (Figure 5c). The differentiated CSCs showed significant increases in stretching and human epidermal growth factor receptor 2 (HER2) expression when compared to their undifferentiated counterparts (Figure 5(ci,cii)). CSCs were then mixed with the optimized hydrogel block and cultured in media. After 3 days, the cells began to form a spheroid structure, and were seen migrating out to surrounding areas after 6 days of culture (Figure 5(ciii)). Subsequently, the breast CSCs were injected into the previously described 3D neural network model, and followed by monitoring the interactions between the neural network and cancer (Figure 5d). Analysis showed that a cell cluster formed inside the neural network 3 days after the injection showed an increased expression of synapsin I at the synaptic junctions of the surrounding neural networks [111]. Conversely, the synapsin I signal disappeared from the surrounding neural cell network after 7 days, and a higher expression of the HER2 marker was found in the breast CSC cluster, implying differentiation of the CSCs. It is also likely that the differentiated CSCs induced negative effects on the neural network by disconnecting the synapses via cancer spreading or toxic cytokine secretion [112]. Consequently, our metastatic brain tumor spreading model can be applied as an effective tool for monitoring the cellular response and behavior of metastatic brain cancer and surrounding cells to screen therapeutics.

## 4. Future Directions

To date, most microdevice-based nervous system mimicking and their disease model applications have mainly focused on the representing known mechanisms of the device, and verified isolated observations. By taking this advantage, the final goal of the microdevice-based nervous system is in mimicking the specific disease with patient-derived cells, and utilizing it as a screening tool to find personalized medicine. To realize this goal, the cellular functionality monitoring method needs to be integrated into the brain mimicking microdevice. The current cellular condition or signal monitoring methods on microdevices are heavily weighted on immunofluorescence. But, in most of cases, the immunofluorescence method needs a fixation process, which means that in situ monitoring of neurons is not possible. The method of communication in the nervous system is electrical in its basis, therefore, the functionality of neurons can be monitored by electrical and electrochemical detection of the cellular signals and neurotransmitter secretions [113,114,115,116,117]. 

### 4.1. Electrochemical Detection of Neurotransmitter

**Figure 6:**
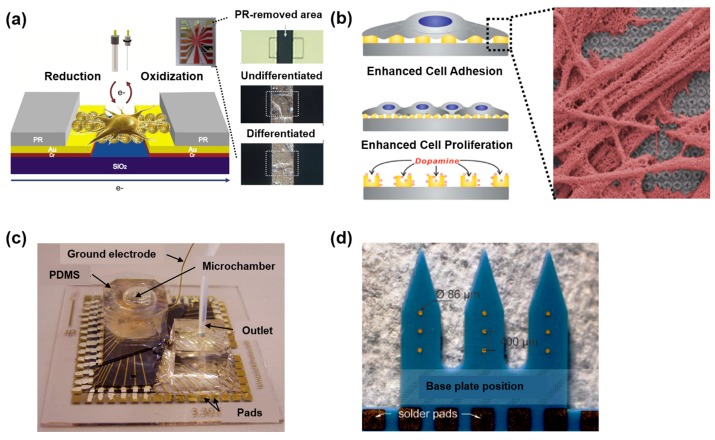
Electrochemical system for the analysis of various nervous signals on the micro-platform. (**a**) Schematic diagram of the electrochemical characteristics of undifferentiated/differentiated single mouse NSC (mNSC). The redox characteristics of single mNSC in two different states can be verified by using this micro gap substrate. Reproduced with permission [100]. Copyright 2013, Elsevier; (**b**) The NSC-based chip consists of large-scale homogeneous nano-cup electrode arrays (LHONA) used for the detection of dopamine released from dopaminergic cells. Reproduced with permission [114]. Copyright 2015, Wiley; (**c**) Multielectrode arrays (MEA) platform for long-term noninvasive assessment of human dorsal root ganglia (DRG) cell health and function. The inset shows the bright field micrograph of electroplated MEA. Reproduced with permission [115]. Copyright 2009, Royal Society of Chemistry; (**d**) The low-temperature cofired ceramics (LTCC) model for the design of a 3D-MEA. Finger conductor circuit with tower electrodes is designed to fit measurement adapters for signal recordings and data processing. Reproduced with permission [122]. Copyright 2015, Göller Verlag GmbH.

Neurotransmitters are key cytokines that enable cellular communication in neural networks. Therefore, neurotransmitter detection is a reliable method for confirmation of the functionality of neural cells and neuronal networks. Dopamine is an attractive target molecule for neuron maturity confirmation, considering its specific electrochemical characteristics [118,119,120]. To quantify the secreted amount of dopamine, researchers have developed in situ detection methods by utilizing ECM modified electrodes [113,114,115,116,117]. Cho et al. have developed arginyl–glycyl–aspartic acid (RGD) peptide modified electrodes by nanopatterning, followed by mixing with conductive materials to improve the bio-affinity and sensitivity of cellular signal detection [117,121]. Kim et al., on the other hand, fabricated metal nanopatterned electrodes with graphene oxide, which can detect both dopamine secretion, as well as an electrochemical signal difference before and after neuronal stem cell differentiation [115,122] (Figure 6a,b). 

### 4.2. Multielectrode Array Based Electrical Signal Detection

Multielectrode arrays (MEAs) have been utilized to confirm the electrical activity of neural cells in neural networks [123,124,125,126,127,128,129,130]. Considering MEAs’ capability for not only the in situ detection of cellular electrical signals, but also electrical stimulation, they are ideal tools to apply to nervous system-on-a-chip systems. Musick et al. have fabricated a multi-layered MEA on microfluidic devices. In this system, a rat cortical neuronal network was formed, and its electrical signal could be recorded up to 28 days simultaneously [123] (Figure 6c). Dworak et al. have guided neuronal axon growth with micro-tunnels on an MEA [124]. The propagated action potential was successfully detected by the MEA as single axons grew and passed multiple electrodes. Abbott et al. have created a nanoelectrode array for intracellular electrophysiological imaging with a complementary metal oxide semiconductor (CMOS) [129]. This CMOS nanoelectrode array has needle-shaped vertical nanoelectrodes that can penetrate the cellular membrane, accessing the intracellular environment. Despite these advancements, current MEAs are limited in acquiring electrical cellular signals from 3D neural networks. To overcome this limitation, Bartsch et al. developed a 3D MEA consisting of tower electrodes arrays [130] (Figure 6d). Single tower electrodes are three electrodes vertically aligned with 400 μm gaps, and have the potential to detect the spatially-specific signals of neurospheres. Although there have been several efforts aimed at developing 3D MEAs for 3D neural network signal detection, spatially specific signal detection remains a challenge, due to the promiscuous nature of the neurospheres in forming random connections.

## 5. Conclusions

The development of advanced microdevice fabrication techniques enables the mimicry of in vivo microphysiological conditions, as within an in vitro organ model. Here, we introduce the current approaches of in vitro brain-like microdevice systems. Due to the complexity of the different interconnected cells of the nervous system, various approaches of neural network mimicking systems have been described herein. First, the 2D compartmentalized co-culture system on microdevices allows for the analysis of the interactions between neurons and glial cells in the CNS, such as their communication involving neurotransmitters, and the axonal growth control of neurons. Second, the fabricated 3D neural network not only allows the simulation of the 3D structure of brain tissues (such as the cortex) but also replicates the modular interaction between the cortex and the hippocampus. Third, the neurovascular mimicking model can show the interplay of the microvascular endothelium and its secretomes on the nervous system, as well as the influence of leukocytes from blood vessels. These approaches also show their ability to reconstitute in vitro brain disease models, such as TBI, neuroinflammatory, and neurodegenerative diseases.

In the near future, it is anticipated that advances in reconstructing human microenvironmental systems, using human-derived cells and analysis methods, will lead to powerful in vitro systems that can minimize the gap between pre-clinical and clinical research in the biomedical fields. The high throughput screening of brain diseases with nervous system-on-a-chip will help to address the underlying mechanisms, and facilitate the development of more effective therapies. 

## Figures and Tables

**Table 1:** Advantages and limitations of current central nervous system model on a microdevice.

Condition			Advantage	Limitation	Function	Ref.
**2D neural network**	Tissue culture plate-based co-culture		Simple structure Easy to use	Randomly established connections with other types of neural cells Overlapping cellular responses	Monitoring cell–cell interaction	[23,24,25]
	Horizontally-aligned neural network		Axonal growth direction control Compartmented structure for spatial drug treatment	Not applicable for the deep tissue drug diffusion	Monitoring neuron–glial neuron–neuron interaction	[26,27,28,29]
	Vertically-aligned neural network		In situ collection of cytokines	No physical interaction between different cell layer Long chip preparation time Not able to treat chemical on specific layer of cell Not applicable for the deep tissue drug diffusion	Secreted cytokine based cellular communication	[30]
**3D neural network models**	Hydrogel-based 3D neural network	Individual cell	Monodispersed neural network	Contraction of hydrogel Different axonal growth with different stiffness of hydrogel	3D neural signal monitoring	[31,32,33]
		Spheroid	Novel 3D, spontaneously active networks Brain-approximating characteristics	Contraction of hydrogel Limited spheroid size Not able to compartmental control to study cell-specific features	Monitoring the developmental process of brain in vitro	[34,35]
	Gel-free 3D neural network	Spheroid	Mimicking the interaction between different region of brain	Limited spheroid size Easy to spread the cell after attaching on substrate	Visualization of the spatiotemporal morphological changes of single neurons	[36,37]
		Scaffold	Compartmented structure formation Mechanical stability Easy to handle High diffusion rate with porous structure	Making monodispesed cell condition in the scaffold	Mimicking the cerebral cortex Brain homeostasis and injury study	[38,39]
**Neurovascular unit models**	Horizontally-aligned neurovascular models		Making tight junction structure Ease to see drug permeability change the trans-endothelial electrical resistance (TEER) within a short time Dividing apical and basolateral space to co-culture the endothelium and astrocyte	The discrepancy with in vivo vascular flow and shear stress Not presenting neuronal cells The difference of the connecting material between the cells with the actual membrane Not inducing polarization of vascular endothelium and its interaction to astrocyte	Testing drug permeability reactive oxide species (ROS) generation by hydrogen peroxidase and confirmation of the change TEER value Enhancement of the efflux by upregulating P-gp. Disruption and recovery of the barrier function	[40,41,42]
	Vertically-aligned neurovascular models		Mimicking the actual shear stress The interaction between endothelium and astrocyte and/or pericyte Monitoring the TEER value Insertion of the 3D extracellular matrix (ECM) materials into the chip	Not presenting whole neurovascular unit cells. Absorption of the hydrophobic molecules to the channel and membrane The difference of the connecting material between the cells with the actual membrane	Permeability test using fluorescein isothiocyanate (FITC) conjugated small molecule Enhancement of blood–brain barrier (BBB) integrity compares of Transwell system Change the TEER value for several stimuli such as histamine, glutamine and tumor necrosis factor alpha (TNF-α)	[43,44,45,46,47]

**Table 2:** Advantages and limitations of current disease models on a microdevice.

Disease Model		Advantage	Limitation	Function	Ref.
**Neurodegenerative disease models**	Axonal injury models	Easy to mimic the damaged state to axon Induction of axonal growth direction Facilitates biochemical analyses, such as chemical treatment	Discrepancy of the actual neurodegenerative disease Not presenting neurovasculatures and their interactions Not using the 3D ECM materials	Disconnection and regeneration of the axon using simple methods Myelination of the oligodendrocyte along with axonal growth	[91,92,93]
	Alzheimer’s disease models	Simple to induce the Alzheimer’s disease (AD) model by applying Aβ Monitoring of the cell viability and physiological alteration by applying Aβ	Not emulating the interaction between neuronal cells and vascular cells during AD progression Short maintainence period when comparing Aβ deposition time	Analysis of neuronal cell viability by applying Aβ Microglia migration assay by applying Aβ Mimicry the interstitial flow in the brain	[62,94]
Neuroinflammation models		Appropriated shear stress to the endothelium with neuronal cells Observation of change the BBB permeability and neuronal viability simultaneously with intercellular interaction	Some missing components, such as pericytes, astrocytes, microglia and monocytes Focusing on the BBB integrity, lack of neuronal function and viability	Change the TEER value of the BBB by neuroinflammation Analyze the detailed mechanism and metabolism of the neuroinflammation	[95,96,97]
Metastatic brain tumor model		Simultaneous observation of interaction between cancer and surrounding neuron	Limited size to tumor growth Imaging of target cell No angiogenesis	Monitoring of metastatic cancer spreading/migration Monitoring of interaction between neural network and cancer cells	[98,99,100]

## Abbreviations

BBB, blood-brain barrier; CNS; central nervous system, 3D, three-dimensional; PNS, peripheral nervous systems; 2D, two-dimensional; ECM, extracellular matrix; NSCs, neural stem cells; ESCs, embryonic stem cells; iPSCs, induced pluripotent stem cells; PDMS, polydimethylsiloxane; PLL, poly-l-lysine; NBB, neural building block; PLLA, poly-l-lactic acid; PGA, polyglycolic acid; PLGA, poly-lactic-co-glycolic acid; HUVEC, human umbilical vein endothelial cell; P-gp, P-glycoprotein; TEER, trans-endothelial electrical resistance; FITC, fluorescein isothiocyanate; TNF-α, tumor necrosis factor alpha; ICAM-1, intercellular adhesion molecule 1; BDNF, brain-derived neurotrophic factor; NT3, neurotrophin-3; EGTA, ethylene glycol-bis(β-aminoethyl ether)-*N*,*N*,*N*’,*N*’-tetraacetic acid; TBI, traumatic brain injury; AD, Alzheimer’s disease; Aβ, amyloid-beta; G-CSF, granulocyte colony-stimulating factor; LPS, lipopolysaccharide; IL-6, interleukin 6; IL-1β, interleukin 1β; MCP-1, monocyte chemoattractant protein 1; MCP-2, monocyte chemoattractant protein 2; MAP2, microtubule-associated protein 2; CSC, cancer stem cell; HER2, human epidermal growth factor receptor 2; RGD, arginyl–glycyl–aspartic acid; MEA, Multielectrode array CMOS, complementary metal-oxide–semiconductor.
